# Defining ‘Normal’ Liver Function Tests

**DOI:** 10.7759/cureus.97561

**Published:** 2025-11-23

**Authors:** Priscilla Chong, Dhanushan Gnanendran, Yu Xiang Gao-Du, Charles Millson

**Affiliations:** 1 Gastroenterology, York and Scarborough Teaching Hospitals NHS Foundation Trust, Scarborough, GBR

**Keywords:** chronic liver disease, diagnostic limitations, early detection, lft - liver function tests, liver cirrhosis

## Abstract

Background

Chronic liver disease often remains undiagnosed until cirrhosis develops, with standard liver function tests (LFTs) sometimes appearing normal despite significant hepatic dysfunction.

Aim

To investigate and analyze index liver function tests (LFTs), hematological parameters, and secondary care clinical correlates in patients with confirmed cirrhosis to better understand the clinical utility of LFTs.

Methods

A retrospective, single-center observational study was conducted on 251 randomly selected patients from a total of 563 with confirmed cirrhosis. Diagnosis was confirmed radiologically or histologically. Patients with clinically evident liver disease were excluded. Data included LFTs, full blood count, international normalized ratio (INR), and albumin-bilirubin (ALBI) score. Subgroup analysis was performed comparing those with normal vs. abnormal LFTs.

Results

Of the 251 patients, 37% had ‘normal’ LFTs, increasing to 48% among those without clinical correlates. The aspartate aminotransferase (AST): alanine transaminase (ALT) ratio was 1.5 for the full cohort and 1.29 for those with normal LFTs. The ALBI score was normal in 59.1% of the total cohort and in 83.3% of the normal LFT subgroup. These findings indicate that standard LFTs alone may miss a significant proportion of cirrhotic patients, particularly those with compensated disease.

Conclusion

A substantial proportion of cirrhotic patients presented with normal LFTs, highlighting the limitations of relying solely on these tests for diagnosis. The use of additional clinical indicators and non-invasive tools may enhance early detection. These findings underscore the need for a more comprehensive and multi-parametric approach in evaluating and managing cirrhotic patients.

## Introduction

Chronic liver disease (CLD) represents a growing health concern worldwide [1]. As one of the leading causes of premature mortality, CLD poses significant challenges to public health and healthcare systems [2]. The insidious nature of liver disease progression, often asymptomatic until advanced stages, underscores the critical importance of early detection and intervention [3]. The global burden of liver disease is significant, with non-alcoholic fatty liver disease (NAFLD) affecting around 25-38% of the world's total population. This condition is now the leading cause of chronic liver disease worldwide. About 2-3% of individuals with NAFLD progress to cirrhosis, which is a major contributor to liver-related morbidity and mortality [4].

In clinical practice, liver function tests (LFTs) - a panel of biochemical assays performed on a blood sample to evaluate the liver’s metabolic, synthetic, and excretory functions - remain the cornerstone of the initial assessment when liver disease is suspected. Such diseases encompass a broad spectrum of conditions, including viral hepatitis (hepatitis B and C), alcohol-related liver disease, NAFLD, autoimmune hepatitis, drug-induced liver injury, cholestatic disorders (e.g., primary biliary cholangitis and primary sclerosing cholangitis), and metabolic or genetic liver diseases such as hemochromatosis and Wilson’s disease [5]. LFTs typically include measurements of bilirubin, albumin, alanine aminotransferase (ALT), and alkaline phosphatase (ALP) [6,7]. These biochemical markers provide insight into different aspects of hepatic function, specifically, the liver’s synthetic capacity, excretory ability, and hepatocellular integrity [7]. However, the interpretation of LFT results, particularly when values fall within the ‘normal’ range, warrants careful consideration [8].

The concept of a normal LFT is deceptively simple yet potentially misleading. Laboratory reference ranges are typically established based on population statistics, often representing the central 95% of values observed in apparently healthy individuals [9]. However, these ranges may not adequately capture the nuanced changes that occur in liver function during the early stages of the disease or in compensated chronic liver conditions [9]. Consequently, relying solely on LFTs to rule out significant liver pathology may lead to missed opportunities for early intervention and management.

The progression of liver disease to cirrhosis represents a critical juncture in the natural history of CLD. Cirrhosis, characterized by extensive fibrosis and distortion of liver architecture, can have profound impacts on overall liver function and patient outcomes [10]. However, the relationship between cirrhosis and LFT results is not straightforward. Compensated cirrhosis may present with minimal alterations in conventional LFT parameters, potentially leading to false reassurance if these tests are interpreted in isolation [11].

It is noteworthy that medical education often emphasizes the need for referral to hepatology services, primarily in cases of abnormal LFTs. This approach is reinforced by numerous clinical guidelines that use abnormal LFTs as a starting point for further investigation, implicitly suggesting that normal LFTs equate to normal liver function [8]. For example, the National Institute for Health and Care Excellence (NICE) - a UK body that develops evidence-based recommendations to improve healthcare quality and outcomes - issues guidelines that adopt this framework. The NICE guideline NG50 on cirrhosis in adults over 16 years begins its care pathway with the identification of abnormal LFTs [12]. Similarly, the NICE guideline NG49 on the assessment and management of NAFLD focuses primarily on patients presenting with abnormal LFT results [13].

Given the limitations of LFTs in detecting early or compensated liver disease, there is a pressing need to re-evaluate our approach to liver disease screening and diagnosis [5]. This study aims to address this gap by examining the prevalence of normal LFTs in a known cirrhotic population. By focusing on patients with confirmed cirrhosis, we seek to challenge the conventional wisdom surrounding the interpretation of normal LFT results and highlight the potential for missed diagnoses when relying solely on these tests.

Our study extends beyond standard LFTs to include full blood count (FBC), aspartate aminotransferase (AST), and international normalized ratio (INR), clinical correlates (bleeding varices, ascites, hepatic encephalopathy), and prognostic indicators (Child-Pugh status, ALBI score). This comprehensive approach provides a more complete view of liver function and associated changes.

The primary aim is to determine the proportion of confirmed cirrhotic patients with normal LFTs and highlight the limitations of conventional LFT interpretation.

## Materials and methods

Study design and patients

This was a retrospective observational study conducted at York and Scarborough NHS Foundation Trust. Patients were identified from the departmental cirrhosis database, which includes individuals with a confirmed diagnosis of cirrhosis based on clinical, laboratory, imaging, and/or histological findings. A total of 563 patients with confirmed cirrhosis were available in the database during the study period. All data were anonymised prior to analysis to maintain patient confidentiality, and the study adhered to the principles of the Declaration of Helsinki.

The data used in this study were accessed for research purposes on September 12, 2024.

The authors did not have access to information that could identify individual participants during or after data collection. Data were stored and analysed using encrypted NHS-approved platforms to ensure data protection and information governance compliance.

The study population was derived from a departmental cirrhosis database, which contains all those with a confirmed diagnosis of cirrhosis via ultrasound or computer tomography imaging, and/or histological findings over a period of three years from 2020 to 2023. A total of 563 patients with confirmed cirrhosis were available in the database during the study period at York and Scarborough, linked to hospital admission and GP prescribing data. LFTs included bilirubin, albumin, alkaline phosphatase (ALP), alanine transaminase (ALT), and AST. To minimize selection bias and ensure a representative distribution of patient characteristics and disease etiology, a stratified random sampling method was used to extract 251 patients using a computer-generated randomization.

Patients aged 18 and above with no obvious or reported clinical signs of a liver disease on presentation to their GP, and with LFTs requested at the index GP appointment between 2020 and 2023, were eligible for inclusion. The following exclusion criteria ensured that the study population of patients had no clinically recognised liver disease at presentation in primary care: Bilirubin greater than 35 µmol/L at baseline, suggesting jaundice; Diagnosis of ascites, encephalopathy, varices, or portal hypertension within 6 weeks of their first LFTs; History of any liver disease before baseline.

To investigate correlations between LFTs and the severity of liver disease progression leading to cirrhosis, secondary parameters within secondary care were assessed. Clinical indicators of cirrhosis were defined as follows: bleeding varices were included only if confirmed by endoscopic findings, radiologically confirmed ascites, and hepatic encephalopathy was documented only if clinically assessed by a hepatologist.

Exclusion criteria included patients with incomplete laboratory data or missing key clinical information, those with a history of liver transplantation, active cancer, and individuals experiencing acute liver failure or decompensated liver disease that required immediate intervention.

Data collection

Data were extracted from electronic medical records, including demographic information (age, gender), laboratory values, clinical correlates, and liver disease severity scores.

The primary variables comprised LFTs, including bilirubin, albumin, ALT, and ALP. FBC parameters, such as hemoglobin, white blood cells, and platelet count, were also collected, alongside coagulation parameters, particularly INR. AST data were available for a subset of 117 patients.

The nLFT group was composed of patients whose entire panel of standard liver function tests (LFTs), including serum albumin, ALP, ALT, and bilirubin, fell within the normal reference ranges established by the laboratory. Importantly, all these parameters had to be simultaneously within their respective normal limits for a patient to be classified as having nLFTs; if any single parameter was outside the reference range, that patient was not included in the nLFT subgroup.

The secondary variables included clinical correlates of cirrhosis, such as the presence of bleeding varices, ascites, and hepatic encephalopathy. Additionally, liver disease severity scores like the Child-Pugh status and the albumin-bilirubin (ALBI) score were recorded. The AST: ALT ratio was also assessed to gauge disease progression.

Statistical analysis

Data were analysed using SPSS software (IBM Corp., Version 27.0, Armonk, NY, US) with descriptive and comparative analyses. Continuous variables were summarized as mean (SD), and categorical variables as frequencies and percentages. Comparative analyses assessed the proportion of patients with normal LFTs overall and stratified by clinical correlates of cirrhosis. Laboratory values (albumin, ALP, ALT, bilirubin, AST, INR) were compared between normal and abnormal LFT groups using one sample independent t-test, comparing the mean values of each parameter to the upper limit of the laboratory normal ranges (and lower limit for the albumin), with effect sizes measured using Cohen's d and Hedges' correction for standardization and cross-metric comparison. A p-value <0.05 indicated statistical significance. This study was conducted in accordance with the ethical principles of the Declaration of Helsinki [14]. Patient confidentiality was maintained by anonymizing all data during extraction and analysis.

## Results

This study analyzed data from 251 randomly selected patients with confirmed cirrhosis from a departmental database of 563 cases. Cirrhosis aetiology was attributed to metabolic dysfunction-associated steatotic liver disease (MASLD)/metabolic dysfunction-associated steatohepatitis (MASH) in 35% of patients, alcohol-related liver disease in 30%, viral hepatitis in 20%, autoimmune or cholestatic disorders in 10%, and other or cryptogenic causes in the remaining 5%. The analysis focused on LFTs, full blood count, coagulation parameters, and clinical features.

Of the 251 patients, 89 (37.0%) had nLFTs. A total of 143 patients (57.1%) had a normal platelet count, and 55 (22.4%) had a normal INR. Clinical correlates of cirrhosis, such as bleeding varices, ascites, or encephalopathy, were absent in 171 patients (68.0%), classifying them as Child-Pugh A. Among these 171 patients without clinical correlates, 82 (48.0%) had normal LFTs.

To better understand clinical and biochemical differences, patients were divided into two groups: those with normal LFTs (nLFT) and those with abnormal LFTs (abnLFT). The mean age was similar between groups (66.1 vs. 65.1 years, p = 0.43), as was the proportion of male patients (57.6% vs. 59.7%, p = 0.83). MASLD/MASH was slightly more prevalent in the nLFT group (41.2%) than in the abnLFT group (34.3%), though this was not statistically significant (p = 0.15).

Among laboratory values, ALT was significantly lower in the nLFT group (38.5 vs. 42.7 IU/L, p = 0.03). No statistically significant differences were found for albumin, ALP, bilirubin, or ALBI score. These findings suggest that standard clinical and biochemical parameters may not reliably differentiate between patients with and without abnormal LFTs in cirrhosis. Full results are presented in Table 1.

**Table 1:** nLFT vs abnLFT nLFT: normal liver function test; abnLFT: abnormal liver function test; ALP: alkaline phosphatase; ALT: alanine transaminase; ALBI: albumin-bilirubin; MASLD: metabolic dysfunction-associated steatotic liver disease; MASH: metabolic dysfunction-associated steatohepatitis

Parameter	nLFT (Mean ± SD or %)	abnLFT (Mean ± SD or %)	p-value
Age	66.13 ± 9.92	65.08 ± 10.81	0.427
Albumin (g/dL)	42.10 ± 5.09	42.38 ± 4.65	0.658
ALP (IU/L)	138.23 ± 34.74	134.18 ± 32.84	0.351
ALT (IU/L)	38.47 ± 14.89	42.66 ± 14.84	0.029
Bilirubin (mmol/L)	34.14 ± 9.68	34.68 ± 10.12	0.672
ALBI Score	-2.57 ± 0.42	-2.50 ± 0.41	0.444
Male (%)	57.6%	59.7%	0.831
MASLD/MASH (%)	41.2%	34.3%	0.153

AST: ALT ratio and ALBI score analysis

The AST: ALT ratio was calculated for the 117 patients with available AST data. The mean ratio for the entire cohort was 1.5 (SD 0.45), consistent with cirrhotic profiles but not always raising clinical suspicion. Among patients with normal LFTs, the AST: ALT ratio was slightly lower at 1.29 (SD 0.38).

The ALBI score was within the normal range in 148 patients (59.1%) across the whole cohort. In the nLFT subgroup, 77 patients (83.3%) had a normal ALBI score, with a mean score of -2.71 compared to -2.53 for the total population.

## Discussion

Our study highlights critical limitations in the traditional reliance on LFTs for the assessment of cirrhosis. Notably, 37.0% of patients with confirmed cirrhosis had normal LFTs - a figure that increased to 48% in those without overt clinical signs of liver dysfunction. These findings align with previous research demonstrating the limited sensitivity of LFTs in detecting advanced liver disease [11], and they have substantial clinical implications, particularly regarding delayed diagnosis and missed opportunities for early intervention.

The AST: ALT ratio in our cohort was 1.5 overall and 1.29 among patients with normal LFTs. While consistent with chronic liver disease [15], this modest elevation underscores the subtlety of biochemical abnormalities in some cirrhotic patients, which may be overlooked in routine evaluations. Similarly, although the ALBI score is a more refined measure of liver function, it was normal in 59.1% of the cohort and 83.3% of patients with normal LFTs, suggesting that it too may miss cirrhosis in well-compensated patients [16].

Routine laboratory markers, such as platelet count and INR, were frequently normal. Over half (57.1%) of cirrhotic patients had normal platelet counts, and 22.4% had normal INR values. These results likely reflect the liver’s substantial functional reserve and compensatory mechanisms, which can preserve normal lab values despite underlying fibrosis [17].

Multiple factors, including reduced hepatocyte mass limiting enzyme release, fluctuating necroinflammatory activity, advanced fibrosis with diminished enzyme production, and individual variability in metabolic or immune responses, may contribute to the high prevalence of normal LFTs in cirrhotic patients. First, the liver’s regenerative capacity can maintain near-normal function until late stages of the disease [17]. Second, liver enzyme levels fluctuate, and single measurements may not reflect the true disease burden [18]. Third, the aetiology of liver disease influences biochemical profiles; for instance, patients with NAFLD may exhibit minimal enzyme elevations [19]. Finally, standard LFTs predominantly reflect liver injury (e.g., AST, ALT), synthetic capacity (e.g., INR, albumin), or cholestasis (e.g., ALP, gamma-glutamyl transferase (GGT)), but do not directly assess fibrosis or portal hypertension [11].

These findings align with previous work. Newsome et al. reported that up to 50% of patients with cirrhosis had normal ALT levels [8], and Giannini et al. noted that the AST: ALT ratio loses diagnostic value in patients with normal transaminases [20]. The discrepancy in thrombocytopenia prevalence between our study (42.9%) and that of Qamar et al. (76%) may be due to differences in disease stage or patient demographics [21].

The ALBI score's performance in our cohort warrants careful interpretation. While Johnson et al. demonstrated its utility in prognosticating hepatocellular carcinoma [22], its reduced sensitivity in identifying cirrhosis among patients with normal LFTs highlights potential limitations in broader clinical use.

We also observed a notable correlation between bleeding varices and certain laboratory abnormalities, particularly low platelet count and elevated AST levels. This is consistent with previous findings by Berzigotti et al., who identified thrombocytopenia as a predictor of esophageal varices in cirrhotics [23]. Moreover, our findings also emphasized the importance of non-invasive markers, such as platelet count and AST, in predicting portal hypertension and variceal bleeding [24]. Our data extend these observations by demonstrating similar associations even in patients with normal LFTs, highlighting their potential utility in risk stratification.

These observations collectively challenge the conventional interpretation of normal LFTs as indicative of a healthy liver. Clinicians should be cautious in using LFTs as a sole screening tool, particularly in high-risk populations such as those with metabolic syndrome, alcohol use, or chronic viral hepatitis [3,11]. The term “liver function tests” itself may be misleading, as these assays primarily reflect indirect and static indicators of liver injury or synthesis and are subject to variability due to intercurrent illness, biliary obstruction, or vitamin K deficiency.

Moving forward, a more comprehensive approach to liver disease detection is warranted. Non-invasive assessment tools, such as transient elastography (FibroScan) - an ultrasound-based technique that measures liver stiffness, and non-invasive serum-based fibrosis scores (e.g., the fibrosis-4 (FIB-4) index and the AST to platelet ratio index (APRI)) should be considered in primary care, particularly when clinical suspicion persists despite normal LFT results [25]. These methods demonstrate greater sensitivity for the detection of hepatic fibrosis and early cirrhosis.

Emerging technologies may also play a critical role. Artificial intelligence-based decision support tools that integrate patterns from routine labs, imaging, and patient risk profiles could significantly enhance diagnostic accuracy [6]. Additionally, dynamic markers, such as temporal changes in the AST: ALT ratio or postprandial enzyme shifts, might offer further diagnostic insight and warrant exploration in future studies.

Our study’s strengths include its exclusive focus on a confirmed cirrhotic population and the comprehensive evaluation of multiple clinical and biochemical parameters, including ALBI scores and the presence of portal hypertension-related complications. However, limitations include its retrospective and single-center design, which may limit generalizability and introduce selection bias. The absence of longitudinal data and a non-cirrhotic control group further limits our ability to assess progression or compare prevalence across disease stages.

## Conclusions

A substantial proportion of cirrhotic patients in our study had normal LFTs, underscoring that normal enzyme levels do not exclude advanced liver disease. These findings reinforce the need for more comprehensive diagnostic strategies beyond traditional LFTs to ensure the timely detection and management of cirrhosis.
